# Effects of Tocotrienols on Insulin Secretion-Associated Genes Expression of Rat Pancreatic Islets in a Dynamic Culture

**DOI:** 10.3389/fphar.2016.00291

**Published:** 2016-08-30

**Authors:** Ling L. Chia, Ibrahim Jantan, Kien H. Chua, Kok W. Lam, Kamal Rullah, Mohd F. M. Aluwi

**Affiliations:** ^1^Drug and Herbal Research Center, Faculty of Pharmacy, Universiti Kebangsaan Malaysia, Kuala LumpurMalaysia; ^2^Department of Physiology, Faculty of Medicine, Universiti Kebangsaan Malaysia, Kuala LumpurMalaysia

**Keywords:** tocotrienols, insulin, microfluidic system, gene expression profiling, qRT-PCR, molecular docking

## Abstract

Tocotrienols (T3) are well-known for their antioxidant properties besides showing therapeutic potential in clinical complications such as hyperlipidemia induced by diabetes. The aim of this study was to determine the effects of δ-T3, γ-T3, and α-T3 on insulin secretion-associated genes expression of rat pancreatic islets in a dynamic culture. Pancreatic islets freshly isolated from male Wistar rats were treated with T3 for 1 h at 37°C in a microfluidic system with continuous operation. The cells were collected for total RNA extraction and reverse-transcribed, followed by measurement of insulin secretion-associated genes expression using quantitative real-time polymerase chain reaction. Molecular docking experiments were performed to gain insights on how the T3 bind to the receptors. Short-term exposure of δ- and γ-T3 to pancreatic β cells in a stimulant glucose condition (16.7 mM) significantly regulated preproinsulin mRNA levels and insulin gene transcription. In contrast, α-T3 possessed less ability in the activation of insulin synthesis level. Essentially, potassium chloride (KCl), a β cell membrane depolarising agent added into the treatment further enhanced the insulin production. δ- and γ-T3 revealed significantly higher quantitative expression in most of the insulin secretion-associated genes groups containing 16.7 mM glucose alone and 16.7 mM glucose with 30 mM KCl ranging from 600 to 1200 μM (*p <* 0.05). The findings suggest the potential of δ-T3 in regulating insulin synthesis and glucose-stimulated insulin secretion through triggering pathway especially in the presence of KCl.

## Introduction

Insulin is the most potent anabolic hormone secreted by the pancreatic islets of Langerhans of β cell. Insulin functions as a glucose homeostasis regulator in the regulation of insulin gene transcription and translation, preproinsulin mRNA stability control and insulin secretion (Shulman, 2000; Ren et al., 2007). Failure of the body to produce or response to insulin leads to the development of diabetes mellitus. The chronic complications of diabetes mellitus lead to a progressive deterioration in the function of pancreatic β cell and development of hyperglycemia (Bösenberg and van Zyl, 2008) with long-term clinical problems and onset of chronic complications (Niu et al., 2008). Recently, tocotrienols (T3) have been actively investigated due to their therapeutic impacts toward the secondary complications of diabetes mellitus.

**FIGURE 1:**
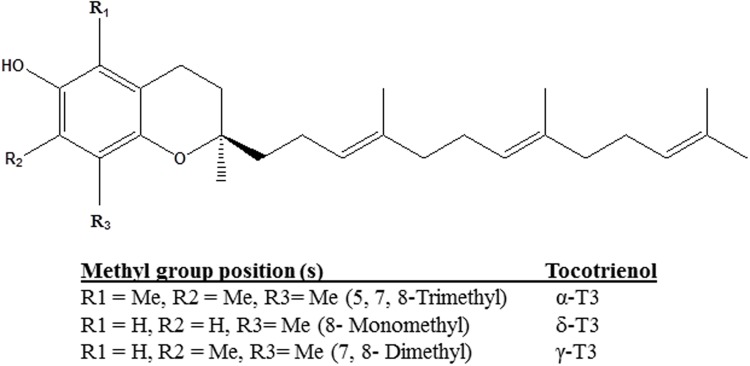
**Chemical structures of tocotrienol isomers**.

Tocotrienols (T3) are naturally occurring derivatives belonging to the vitamin E family synthesized by plants. α-, δ-, and γ-T3 are members of the four unsaturated analogs of vitamin E (Aggarwal et al., 2010). The chemical structures of T3 are slightly different from tocopherols, another category of vitamin E, in the degree of saturation in their farnesyl side chain at the C-2 position. T3 consist of lipid-soluble farnesyl side-chain that provides the added advantage for the differential membrane distribution and metabolism of T3 as compared to tocopherols (Theriault et al., 1999; Vignini et al., 2011). In contrast to their structural similarity, the methyl group position determines the individual compound (**Figure 1**) (Passwater, 2012). The unsaturated chain of T3 is able to penetrate into saturated fatty layer tissues as a significant level of T3 was detected in the brain and liver (Passwater, 2012; Ahsan et al., 2014). Studies had proven that T3 also functioned as signaling molecule in antioxidant properties with α-T3 having 40–60 times more potent antioxidative protection than α-tocopherol in rat liver lipid (Serbinova et al., 1991; Tan, 2005).

The introduction of microfluidic technology in cell culture and pharmaceutical industries provided a platform to greatly improved throughput as well as eliminating the limitations of cell-based studies. A microfluidic device is designed for the long-term monitoring of the cell culture processes combining the typical cell culture practices on an independent microfluidic system. The continuous operation of microfluidic system was able to maintain a stable cell culture environment, with a platform for wide-ranging assays (Hung et al., 2005). The cell culture array offered many potential applications in drug screening, eliminating the limitations in cell-based drug screening assays such as high cost of operation as well as allowing the continuous monitoring of cell culture over long periods of time. The ability of microfabrication technologies to control cell shape, intracellular contact and co-culture interactions are aiding the induction of cells to model their *in vivo* gene expression and functional states more faithfully in culture system and drug discovery (Bhadriraju and Chen, 2002).

While extensive researches has been carried out using tocotrienol rich fractions (TRFs) and T3 on complications of diabetes mellitus, putative effects of each single T3 have not been tested yet on pancreatic β cells. Therefore the present study was carried out to investigate the effects of T3s (δ-, γ-, and α-) on insulin secretion-associated genes expression of normal rat pancreatic islets cultured in glucose and KCl using a microfluidic system.

## Materials and Methods

### Tocotrienols

δ-Tocotrienol, γ-tocotrienol, and α-tocotrienol were purchased from Cayman Chemical, i-DNA (M) Sdn. Bhd, Malaysia (Cayman Chemical, USA). The purity of each T3 was ≥98%.

### Animals

Male adult Wistar rats of age more than 12 weeks old, weight between 300 to 350 g were used in the study. The rats were purchased from the Laboratory Animal Resource Unit (LARU) of Universiti Kebangsaan Malaysia (UKM). The animals were maintained under the guideline of UKM Animal Ethics Committee (UKMAEC). They were kept under standard conditions at room temperature with free access to food and water at all time before use. The use of animals for this study has been obtained the UKM animal ethnic approval of FF/2011/IBRAHIM/19-MAY/371-MAY-2011-AUGUST-2013-AR-CAT2.

### Isolation and Culture of Rat Pancreatic Islets

Rat pancreatic islets isolation was carried out according to Shewade et al. (1999) with minor modifications. Briefly, the animals were anesthetised by anesthetic agent, ilium xylazil-20 (Troy Lab. Pty. Lmt. AUS; 20–40 mg/kg body weight i.v.) which was purchased from LARU of UKM. The pancreas was removed out using aseptic technique, and washed with phosphate buffer saline (PBS; Gibco, Invitrogen, USA) supplemented with antibiotic antimycotic (AA; Gibco, Invitrogen, USA; 2% v/v). Next, enzymatic digestion of the pancreas was carried out with 5 mL of cold collagenase V [0.3% w/v collagenase V (Sigma-Aldrich, St. Louis, MO, USA), 2% v/v AA and 10% v/v fetal bovine serum (FBS; Gibco, Invitrogen, USA)] in 50 mL conical plastic tube for 15 min at 37°C in water bath shaker until completion of digestion. The digestion was terminated by adding 20 mL of PBS and centrifuged at 1000 rpm for 10 min. After digestion, the islets were cultured in supplemented RPMI-1640 (Gibco, Invitrogen, USA) medium (10% v/v FBS, 1% v/v AA) pH-7.2 in 6-well plate (2 ml per well) and kept at 37°C, 5% CO_2_ incubator (RS biotech, UK) overnight to allow recovery from the isolation procedure.

### Tocotrienol Treatment: Dynamic Incubation

The islets with oblong to spherical shape, smooth surface and a diameter of 100–200 μm were used in this treatment. After 24 h incubation, groups of 20 islets were handpicked under Nikon SMZ645 stereomicroscope (Nikon, Japan) and transferred into the cell culture chambers of Cell Asic^TM^ ONIX microfluidic open top plate (Merck EMD, Millipore Corporation, USA) containing 350 μl of preincubation medium. The preincubation medium consisted of Dulbecco’s phosphate-buffered saline (D-PBS; 10% v/v FBS) with basal 2.8 mM glucose. The microfluidic plate was attached to Onix Microfluidic Perfusion System and Onix Microincubator Controller (CellASIC, Millipore Corporation, USA) with supply of 5% CO_2_ at 37°C. The cell culture chambers were also constantly perfused with preincubation medium applied with a flow driven by gas pressure system at 4 psi for 30 min establishing basal levels of secretion. Each group of islets were then treated with 350 μL of different concentrations of T3s (α, γ, and δ; Cayman Chemical, USA) treatment mediums that were perfused at 4 psi for 1 h. The final concentrations of T3s in the treatment mediums (D-PBS with 10% v/v FBS) were 150, 300, 600, and 1200 μM in glucose alone (2.8 and 16.7 mM, as basal and stimulant, respectively; Wicksteed et al., 2003; Ronnebaum et al., 2006; Monfared and Pournourmohammadi, 2010) or together with 30 mM KCl (Sato and Henquin, 1998; Yajima et al., 1999; Shackman et al., 2004). The treated groups were all compared with their respective positive control group (1 μM of glyburide; Kinard and Satin, 1995; Wicksteed et al., 2003) and negative control group (glucose and, or KCl medium only). The β islets were harvested from cell culture chamber for total RNA extraction in the gene expression study.

### Cell Viability Assay

Cell viability assay was carried out with Alamar Blue cell viability reagent (Life Technologies, USA) according to the manufacturer’s standard protocol to assess the survival of treated islets. After 1 h incubation, all T3 treatment groups with 20 islets were transferred into a 96-wells plate. After that, 10% v/v of 10X Alamar Blue cell viability reagent was added into each well-followed by 4 h incubation at 37°C, 5 % CO_2_ incubator (RS biotech, UK). The blue color of resazurin, an active compound of Alamar Blue reagent was converted to red and highly fluorescence resorufin by the viable islets. The absorbance of Alamar Blue was then monitored at 570 nm using 600 nm as a reference wavelength (normalized to the 600 nm value) with Monochromator Microplate (Thermo Scientific, USA). Finally the results were analyzed by plotting absorbance versus T3 concentrations.

### Relative Gene Expression Analysis (RNA Extraction, cDNA Synthesis, Primer Preparation, and qRT-PCR)

#### Total RNA Extraction

Total RNA was extracted from the treated islets group using Tri reagent^®^ (Molecular Research Center, Cincinnati, OH, USA) according to the manufacturer’s recommended protocol. Chloroform was added into the homogenized islets for phase separation followed by isopropanol (Rankem, USA) and polyacryl carrier (Molecular Research Center, Cincinnati, OH, USA) for the precipitation of total RNA forming the RNA pellet. The pellet was then centrifuged at 12,000 rpm for 8 min at 4°C and washed with 75% ethanol (Scharlau, Spain). The pellet was dried for 20 min before solubilising in DNase and RNase free distilled water (Gibco, Invitrogen, USA). The extracted RNA was stored at -80°C for further analysis.

#### Reverse Transcription (cDNA Synthesis)

The total RNA was reversed transcribed using SuperScript^®^ III (Invitrogen, Life Technologies, USA). cDNA was synthesized from 5 μl of total RNA and the reaction was performed according to the manufacturer’s recommended protocol in the following steps: 10 min at 25°C, 30 min at 50°C, 5 min at 85°C followed by 20 min at 37°C. The synthesized cDNA was stored at -20°C for further analysis.

#### Quantitative Real-Time Polymerase Chain Reaction (qRT-PCR)

The synthesized cDNA was used to perform quantitative real-time polymerase chain reaction (PCR) using SYBR^®^ Select master mix to measure the expression level of the insulin secretion-associated genes. Peroxisome proliferator-activated receptors δ (PPARδ), peroxisome proliferator-activated receptors γ (PPARγ), insulin 1 (INS1), glucose transporter 2 (GLUT2), pancreatic/duodenal homeobox-1 (PDX1), V-maf musculoaponeurotic fibrosarcoma oncogene homolog (MafA) and neurogenic differentiation 1 (BETA2) gene expression levels were assessed. Beta-actin (Actb) was used as the housekeeping gene to normalize the data. The specific forward and reverse primers for each gene were designed by using Primer 3 software based on NIH GeneBank database sequences and synthesized by Bio Basic Canada, Inc. (**Table 1**). The PCR reaction was carried out in My iQ-I cycler (Bio-Rad, USA). The reaction mixture consisted of SYBR^®^ Select master mix (Applied biosystem, Life Technologies, USA), forward and reverse primers (500 nM each), deionised water and cDNA. The reactions were carried out in the following parameter: cycle 1: step 1 50°C for 2 min (1 time), cycle 2 (activation of Taq DNA polymerase and pre-denaturation): step 1 95°C for 2 min (1 time), cycle 3 (PCR amplification): step 1 (denaturation): 95°C for 10 s, step 2 (annealing): 56°C for 20 s and step 3 (extension): 72°C for 20 s (50 times), cycle 4: step 1 95°C for 30 s (1 time), cycle 5: 55°C for 1 min (1 time) and cycle 6: 60°C for 10 s (70 times). The relative gene expression level of each gene was obtained by normalizing to Actb. The specificity of the PCR products were confirmed with melting curve analysis following the qRT-PCR protocol and further verified by 2% agarose (Gibco, Invitrogen, USA) gel electrophoresis stained with ethidium bromide (Sigma-Aldrich, St. Louis, MO, USA) and visualized by UV transillumination (Vilber Lourmat, Marne La Vallee, France).

**Table 1:** Description of Insulin secretion associated gene primers used in RT-PCR.

Gene	Accession no	Primer 5′–3′	PCR product size (bp)
PPARδ	NM_013141.2	R 5′-cagcagtccgtctttgttga-3′ F 5′-gatcagcgtgcatgtgttct-3′	193
PPARγ	NM_013124.3	R 5′-gaggccagcatggtgtagat-3′ F 5′-catttttcaagggtgccagt-3′	156
INS1	NM_019129.3	R 5′-ccagttggtagagggagcag-3′ F 5′-gtacctggtgtgtggggaac-3′	200
GLUT2	NM_012879.2	R 5′-cggagaccttctgctcagtc-3′ F 5′-accagctctcctgcagtgtc-3′	191
PDX1	NM_022852.3	R 5′-cgttgtcccgctactacgtt-3′ F 5′-acccgtacagcctacactcg-3′	198
MAF	NM_019318.1	R 5′-ctggttcttctccgactcca-3′ F 5′-aaggaggaggtgatccgact-3′	120
BETA2	NM_019218.2	R 5′-tcttgggcttttgatcatcc-3′ F 5′-ttgaagccatgaatgcagag-3′	120
Actb	NM_031144.2	R 5′-ctctcagctgtggtggtgaa-3′ F 5′-gtcgtaccactggcattgtg-3′	181

### Computational Studies

#### Homology Modeling

The construction of the homology models were initiated with the searching of rat PPARδ and PPARγ amino acids sequences. The amino acids sequences of rat PPARδ (Accession Id: Q62879) and rat PPARγ (Accession Id: O88275) were then retrieved from UniProtKB (The UniProt Consortium, 2009). BLAST program was used to search for the suitable template structures in the Protein Data Bank (PDB) at National Center for Biotechnology Information, NCBI^1^ for the construction of rat PPARδ and PPARγ. Both rat PPARδ and PPARγ exhibit high similarity with human PPARδ (pdb id: 3GZ9; Connors et al., 2009) and PPARγ (pdb id: 1ZGY; Li et al., 2005) with 91.8 and 98.5% of similar identities, respectively. Therefore, these two structures were chosen as templates for model building. Alignment of the amino acids sequences into the abovementioned template crystal structures were performed for both rat PPARδ and PPARγ using the align2d function in EasyModeller 2.0 where 10 3D models of each rat PPARδ and PPARγ were generated (Šali and Blundell, 1993). This improved dynamic programming algorithm was based on a variable gap penalty function that tends to place gaps in solvent exposed and curved regions which decrease the alignment error in standard alignment method. Generated models were further improved through the loop modeling followed by model optimization using the modified version of the Beale restart conjugate gradients method. The model profile plots were generated and analyzed. The lowest DOPE energy of the loaded model was then taken into docking and MD simulation.

#### Molecular Docking

According to the study conducted by Wong and co-workers (Fang et al., 2010), T3s could act as PPAR ligands possibly due to partial structural similarity in comparison of a known PPARγ agonist, troglitazone. Therefore, our group was interested to look at the interactions between α- and δ- T3 and PPAR receptors at atomic level. First the structures of α- and δ- T3 were constructed and energy minimized using CHARMM force field calculation implemented in Accelrys DISCOVERY STUDIO 3.1. The ligands were docked to the plausible binding sites of PPARδ and PPARγ homology models using the in-house CDOCKER protocol. Different conformations were generated for each ligand through high temperature molecular dynamics. The ligands were heated to a temperature of 700 K in 2000 steps followed by 300 K cooling temperature. Then, the ligands were subjected to refinement by grid-based (GRID 1) simulated annealing and full force minimization after random rotation. The ligands were allowed to flex while the protein homology models were held rigid during refinement process. The generated ligand conformations were clustered according to their binding interactions. Finally, the ligand conformation with the highest -CDOCKER interaction energy and -CDOCKER energy was chosen for further analysis and discussion.

#### Molecular Dynamics Simulation

Molecular dynamics simulation was performed with GROMACS 5.0.4 package, employing the GROMOS96 54a7 force field (Van Der Spoel et al., 2005). Protonation states of ionizable residues were chosen based on their most probable state at pH 7.4. The ligand–protein complexes were energy relaxed using the steepest descent energy minimization algorithm. The complexes were then immersed in an octahedron-shaped box with the minimum distance of 2 nm between the protein surface and the box walls. The starting structures were solvated in simple point charge (SPC) water. The system net charge was also neutralized by the adding of Na^+^ counter ions which were randomly substituted by water molecules. MD simulations were performed using the LINCS algorithm (Hess, 2008) to constrain bond lengths and periodic boundary conditions were applied in all directions. Longrange electrostatic forces will be treated using the Fast Particle-Mesh Ewald method (PME; Cerutti et al., 2009). Van der Waals forces and Coulomb potential were treated using a cut-off of 1.4 nm and the simulation time step was set to 2 fs. An initial velocity obtained according to a Maxwell distribution at 300 K is given to all the atoms. During the simulation, Berendsen barostat and thermostat were set at 1 bar and 300 K with a coupling time of τ_P_ = 2 ps and τ_T_ = 0.1 ps, respectively. The production run was set for 10 ns at constant pressure and temperature conditions.

### Statistical Analysis

Student’s paired *t*-test was used to determine the sample size and the results obtained were collected from three samples. All the data was analyzed using Statistical Package for Social Sciences (SPSS, Inc., Chicago, IL, USA) version 17.0. Each treatment group data was presented as mean ± standard error of mean (SEM). The data were analyzed for normality test using Shapiro Wilks statistic and one way analysis of variance (ANOVA) was performed for multiple comparison between the treatment groups with homogeneity test of variances using Tukey *post hoc* test to determine significance difference (*p <* 0.05). *p <* 0.05 was considered to be statistically significant. Student’s paired *t*-test was used to analyze the comparison of insulin gene expression levels of T3 at 150, 300, 600, and 1200 μM.

## Results

The cell viability test was carried out to assess the cytotoxicity level of α-, γ-, and δ-T3 in the 1 h treatment at 150, 300, 600, and 1200 μM in glucose and KCl incubation mediums. All treated cells were viable (>90%) after 1 h incubation (**Table 2**). In the quantitative analysis, data collected from qRT-PCR showed the relative gene expression level of insulin secretion-associated gene, the relationship between the ligand-modulated transcription factors, insulin release gene and insulin gene transcription factor. All the relative gene expressions in T3 treatments were presented as (A) 2.8 mM glucose (basal), (B) 2.8 mM glucose and 30 mM KCl (calcium raising agent), (C) 16.7 mM glucose (stimulant), and (D) 16.7 mM glucose and 30 mM KCl after 1 h incubation (20 islets per group; *n* = 3). Data were normalized to Actb, compared to respective negative control (glucose and KCl buffer only) and positive control (1 μM glyburide; One way ANNOVA *post hoc* Tukey test). Data from qRT-PCR revealed that most of the insulin secretion-associated genes, especially in the stimulant group with KCl showed significantly higher expression in a dose dependent manner when comparing within all four groups of δ- and γ-T3. In contrast, most of the insulin secretion-associated genes expression treated by α-T3 were not significantly affected.

**Table 2:** Cell viability (%) after 1 h tocotrienol treatments.

Tocotrienol	Tocotrienol concentration (μM)	2.8 mM glucose	2.8 mM glucose and 30 mM KCl	16.7 mM glucose	16.7 mM glucose and 30 mM KCl
		Cell viability (%)			
α	150	99.06	97.12	98.4	96.6
	300	97.18	97.36	96.88	94.04
	600	96.6	93.58	91.49	91.99
	1200	94.95	93.77	91.92	90.06
γ	150	98.3	95.5	96.6	97.36
	300	98.75	97.4	96.4	90.55
	600	95.43	96.3	95.3	92.21
	1200	94.5	94.3	94.8	91.67
δ	150	97.76	97.6	96.47	95.41
	300	98.3	94.4	94.21	91.35
	600	97.43	95.6	94.5	90.65
	1200	96.1	93.8	92.21	90.08

### Relative Gene Expression of Ligand-Modulated Transcription Factor

#### PPARδ

**FIGURE 2:**
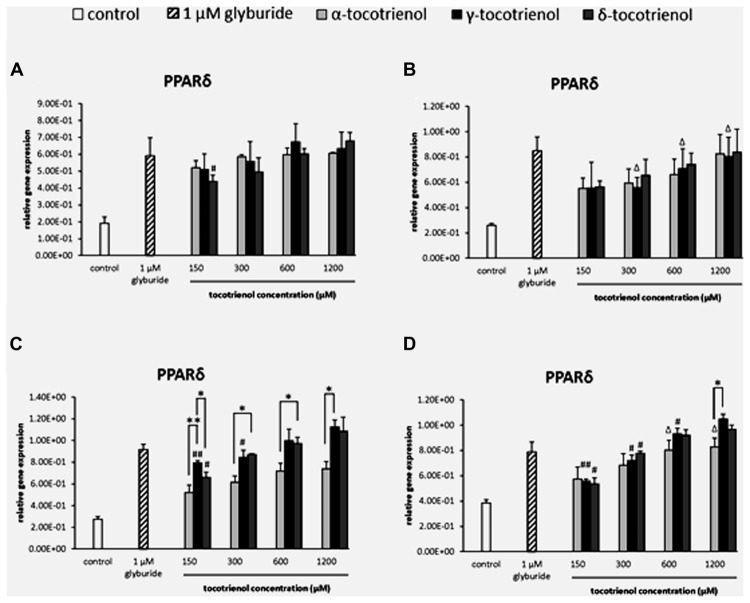
**The relative gene expression level of PPARδ in α-, γ-, and δ-tocotrienol treatment. (A)** 2.8 mM glucose (basal): all α-T3 and δ-T3 groups are 0.01 relative to negative control, all γ-T3 groups are 0.05 relative to negative control, ^#^*p* < 0.05 relative to 1200 μM (same tocotrienol group). **(B)** 2.8 mM glucose and 30 mM KCl (calcium raising agent): all α-T3 and δ-T3 groups are 0.05 relative to negative control, ^Δ^*p* < 0.05 relative to negative control. **(C)** 16.7 mM glucose (stimulant): all α-T3 are 0.05 relative to negative control, all γ-T3 and δ-T3 groups are 0.01 relative to negative control, ^#^*p* < 0.05 and ^##^*p* < 0.01 relative to 1200 μM (same tocotrienol group), *p < 0.05 and **p < 0.01 relative to matched group. **(D)** 16.7 mM glucose and 30 mM KCl: all γ-T3 and δ-T3 groups are 0.05 relative to negative control, ^Δ^*p* < 0.05 relative to negative control, ^#^*p* < 0.05 and ^##^*p* < 0.01 relative to 1200 μM (same tocotrienol group), *p < 0.05 relative to matched group.

The analysis of gene expression showed that PPARδ mRNA was detected in all the groups of T3 treatments compared to the negative control (**Figure 2**). Most of the gene expressions for the T3 treatment groups remained unchanged with no significant difference in the basal glucose conditions (**Figures 2A,B**). In the stimulatory glucose conditions, the effect of α-T3 was less significant whereas significantly higher expressions were observed in δ- and γ-T3 in a dose dependent manner (**Figure 2C**). All T3 treated groups showed significant increase in PPARδ gene expression compared to the negative control. Significant difference (*p* < 0.05) in PPARδ gene expression was observed at 300 and 600 μM when comparing α-T3 to δ-T3. δ-T3 showed significantly more effective treatment (*p* < 0.05) than α-T3 in up regulating the expression of PPARδ by 1.4 fold (*p* < 0.05) at 300 μM and by 1.3 fold (*p* < 0.05) at 600 μM. γ-T3 showed significantly highest PPARδ expression than other T3 groups at 150 μM and significantly higher PPARδ expression as compared to α-T3 at 1200 μM (1.5 fold; *p* < 0.05). In stimulatory glucose with added KCl (**Figure 2D**), the PPARδ gene was expressed significantly greater than the negative control in all four γ- and δ-T3 groups (*p <* 0.05). However, only 600 and 1200 μM of α-T3 showed significant effect on the PPARδ expression compared to the negative control. In δ-T3, a significantly higher expression (*p <* 0.05) was observed at 1200 μM by 1.8 fold compared to 150 μM and by 1.7 fold compared to 300 μM in a dose dependent manner. Whereas in γ-T3, a significantly higher expression was observed at 1200 μM by 1.9 fold compared to 150 μM (*p <* 0.01), by 1.5 fold compared to 300 μM (*p <* 0.05) and by 1.1 fold compared to 600 μM (*p <* 0.05) in a dose dependent manner. The higher concentration of δ- and γ-T3 (1200 μM) in both stimulatory glucose groups also revealed up regulated PPARδ expression comparable to the positive control.

#### PPARγ

**FIGURE 3:**
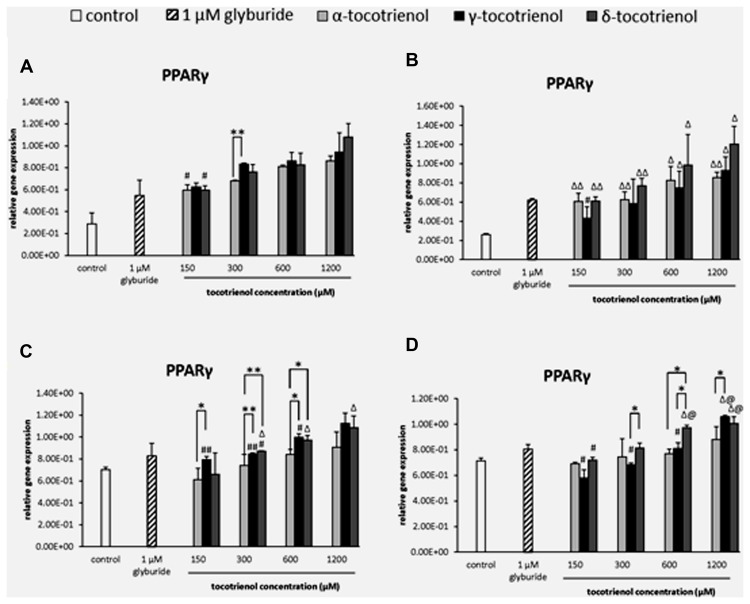
**The relative gene expression level of PPARγ in α-, γ-, and δ-tocotrienol treatment. (A)** 2.8 mM glucose: all tocotrienol groups are 0.05 relative to negative control, ^#^*p* < 0.05 relative to 1200 μM (same tocotrienol group), **p < 0.01 relative to matched group. **(B)** 2.8 mM glucose and 30 mM KCl (calcium raising agent): ^Δ^*p* < 0.05 and ^ΔΔ^*p* < 0.01 relative to negative control, ^#^*p* < 0.05 relative to 1200 μM (same tocotrienol group). **(C)** 16.7 mM glucose (stimulant): all γ-T3 groups are 0.05 relative to negative control, ^Δ^*p* < 0.05 relative to negative control, ^#^*p* < 0.05 relative to 1200 μM (same tocotrienol group), *p < 0.05 and **p < 0.01 relative to matched group. **(D)** 16.7 mM glucose and 30 mM KCl: ^Δ^*p* < 0.05 relative to negative control, ^@^*p* < 0.05 relative to positive control, ^#^*p* < 0.05 relative to 1200 μM (same tocotrienol group), *p < 0.05 relative to matched group.

Similarly, the data from basal glucose groups revealed that most of the T3 groups showed significantly higher expression than negative control, but no dose dependent increase of PPARγ expression was observed indicating that all T3 groups had less impact on PPARγ in both basal glucose groups with low glucose as limiting factor. Only α- and δ-T3 revealed dose dependent increase of PPARγ expression when comparing between 150 and 1200 μM (*p* < 0.05; **Figure 3A**). Whereas in the basal KCl group (**Figure 3B**), all the T3 groups showed significantly higher expression than the negative control, but no dose dependent increase of PPARγ expression was observed indicating that KCl did not give any impact on PPARγ in basal glucose condition. The outcome suggested that in both basal glucose groups, low glucose acted as a limiting factor to PPARγ gene expressions. In the stimulatory glucose groups (**Figures 3C,D**), the expression of PPARγ increased after 1 h of T3 treatment. However, α-T3 effect was inert compared to δ- and γ-T3. δ-T3 treatments indicated a significant upregulated gene expression in a dose dependent manner in stimulatory glucose conditions with a significant PPARγ expression increase by 1.1 fold (*p* < 0.01) compared to α- T3 at 300 μM and by 1.2 fold (*p* < 0.01) compared to α-T3 at 600 μM (**Figure 3C**). While γ-T3 showed a significantly higher gene expression by 1.3 fold (*p* < 0.05) compared to α- T3 at 150 μM, 1.1 fold (*p* < 0.01) compared to α-T3 at 300 mM and by 1.2 fold (*p* < 0.05) compared to α-T3 at 600 μM. The analysis of PPARγ gene expression in the stimulant glucose with KCl group (**Figure 3D**) indicated that KCl further enhanced the expression of PPARγ in δ-T3 stimulatory glucose medium. In δ-T3 treated group, 600 and 1200 μM showed a significant increase (*p* < 0.05) in expression compared to both negative and positive controls. While γ-T3 treatment revealed a significantly upregulated PPARγ gene expression compared to both negative and positive control at 1200 μM (*p* < 0.05). At 1200 μM, γ-T3 treatment also showed a significantly upregulated PPARγ expression as compared to α-T3 (1.3 fold; *p* < 0.05). No significant change was observed in the expression of α-T3 treatment. In δ-T3 treatment, PPARγ expressed in a dose dependent manner with a significant change observed at 1200 μM compared to 150 μM (by 1.4 fold; *p* < 0.05). δ-T3 treatment revealed a significantly higher expression by 1.2 fold compared to γ- T3 (*p* < 0.05) at 300 μM and 1.3 fold compared to α-T3 (*p* < 0.05) at 600 μM, respectively. γ-T3 treatments indicated a significant upregulated gene expression in a dose dependent manner in stimulatory glucose conditions (*p* < 0.05). Whereas δ- T3 revealed the enhancement of the PPARγ gene expression in the stimulatory glucose condition especially in the presence of KCl compared to glyburide indicating that among all three T3s, δ-T3 works most effectively with the presence of KCl in stimulant glucose condition.

### Relative Gene Expression of Insulin Release Gene

#### INS1

**FIGURE 4:**
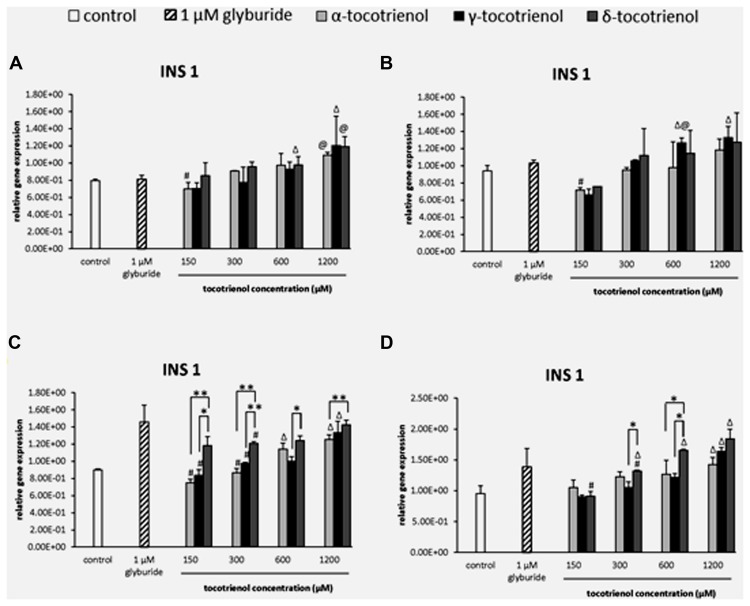
**The relative gene expression level of INS1 in α-, γ-, and δ-tocotrienol treatment. (A)** 2.8 mM glucose: ^Δ^*p* < 0.05 relative to negative control, ^@^*p* < 0.05 relative to positive control, ^#^*p* < 0.05 relative to 1200 μM (same tocotrienol group). **(B)** 2.8 mM glucose and 30 mM KCl (calcium raising agent): ^Δ^*p* < 0.05 relative to negative control, ^@^*p* < 0.05 relative to positive control, ^#^*p* < 0.05 relative to 1200 μM (same tocotrienol group). **(C)** 16.7 mM glucose (stimulant): all δ-T3 groups are 0.05 relative to negative control, ^Δ^*p* < 0.05 relative to negative control, ^#^*p* < 0.05 relative to 1200 μM (same tocotrienol group), *p < 0.05 and **p < 0.01 relative to matched group. **(D)** 16.7 mM glucose and 30 mM KCl: ^Δ^*p* < 0.05 relative to negative control, ^#^*p* < 0.05 relative to 1200 μM (same tocotrienol group), *p < 0.05 relative to matched group.

The effects of T3s in basal glucose groups were minimal in the INS1 gene expression; however, both α- and δ-T3 treatment revealed a significantly higher INS1 expression than glyburide at high dose of 1200 μM (**Figure 4A**). While at 600 μM of basal glucose with KCl (**Figure 4B**), a significant increase of γ-T3 was observed as compared to glyburide. In contrast to basal glucose, stimulatory glucose group (**Figure 4C**) showed better effect of T3 treatment in INS1 expression where all the T3 treated groups upregulated INS1 gene expression in a dose dependent manner in the sequence of effectiveness: α < γ < δ. A significant increase in INS1 expression was observed with δ-T3 treatment at 1200 μM compared to at 300 μM (1.2 fold; *p* < 0.05). δ-T3 showed significantly effective treatment than α-T3 in up regulating the expression of INS 1 at 1200 μM with a significant increase of 1.2 fold (*p* < 0.01) at δ- 1200 μM compared to α-T3 at 1200 μM. A similar effect was observed when KCl was added into the stimulatory glucose group (**Figure 4D**). INS 1 gene was expressed significantly higher than the negative control at α-T3 (1200 μM) and γ-T3 (1200 μM) only while all δ-T3 groups except at 150 μM showed significantly higher INS expression compared to the negative control. δ-T3 revealed significant increase in INS1 expression at 1200 μM compared to 150 μM (twofold; *p* < 0.05) in a dose dependent manner. At 600 μM, δ-T3 showed an increase of 1.3 fold gene expression compared to α- and γ-T3 (*p* < 0.05). The analysis of gene expression showed that glucose played an important role in T3 treated INS1 gene expressions as there was no adverse effect of T3s on the gene expressions in basal glucose groups but significant increase in INS1 expression especially in δ-T3 when incubated in stimulatory glucose condition. KCl in stimulatory glucose further potentiated the effect of δ- T3 in INS1 gene expression.

#### GLUT2

**FIGURE 5:**
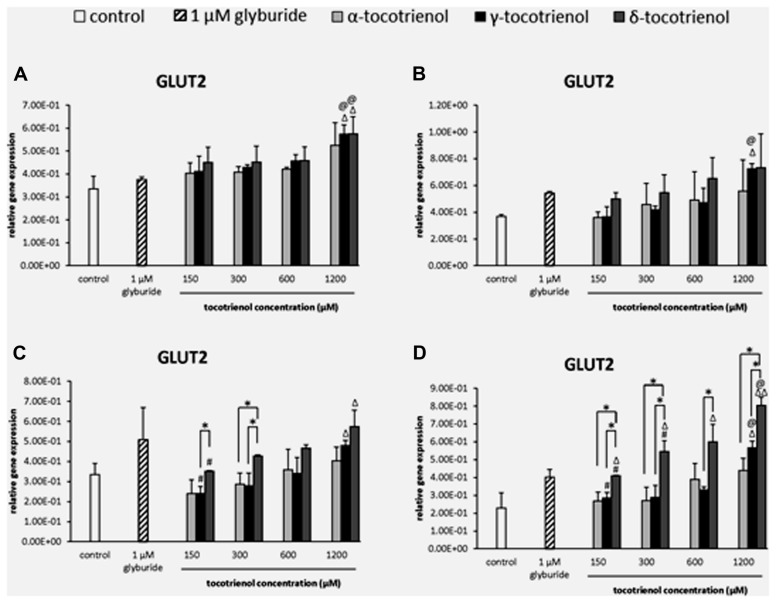
**The relative gene expression level of GLUT2 in α-, γ-, and δ-tocotrienol treatment. (A)** 2.8 mM glucose: ^Δ^*p* < 0.05 relative to negative control, ^@^*p* < 0.05 relative to positive control. **(B)** 2.8 mM glucose and 30 mM KCl (calcium raising agent): ^Δ^*p* < 0.05 relative to negative control, ^@^*p* < 0.05 relative to positive control. **(C)** 16.7 mM glucose (stimulant): ^Δ^*p* < 0.05 relative to negative control, ^#^*p* < 0.05 relative to 1200 μM (same tocotrienol group), *p < 0.05 relative to matched group. **(D)** 16.7 mM glucose and 30 mM KCl: ^Δ^*p* < 0.05 and ^ΔΔ^*p* < 0.01 relative to negative control, ^@^*p* < 0.05 relative to positive control, ^#^*p* < 0.05 relative to 1200 μM (same tocotrienol group), *p < 0.05 relative to matched group.

The effect of T3s on GLUT2 gene expression was dependent on the presence of high glucose especially with δ- T3 revealed a dose dependent increase of GLUT2 expression but not α- T3. In basal glucose (**Figure 5A**) and basal glucose with KCl (**Figure 5B**), the data demonstrated that GLUT2 gene was only able to respond to δ- and γ-T3 treatment in basal glucose or basal glucose with KCl administration at 1200 μM. The effects of δ- and γ-T3 were enhanced in stimulatory glucose showing gradual increase in GLUT2 expression. In stimulatory glucose group, δ-T3 at 1200 μM showed a significant higher gene expression compared to the negative control and a 1.6 fold increase compared to δ-T3 at 150 μM (*p* < 0.05). γ-T3 at 1200 μM also showed a twofold increase in gene expression compared to γ-T3 at 150 μM (*p* < 0.05) while no adverse effect of α-T3 was observed. At 300 μM, δ-T3 produced an increase of 1.8 fold gene expression compared to γ- and α-T3 (*p* < 0.05; **Figure 5C**). In contrast, when the stimulatory glucose incubation medium was added with KCl, the effect of δ- T3 was augmented with all the δ-T3 treated groups showing significantly higher GLUT2 expression compared to the negative control. T3 facilitated the uptake of glucose into β cell with the presence of KCl by further boosted the effect of δ- and γ-T3 on GLUT2 gene expression in a dose dependent manner and better expression compared to α-T3 (**Figure 5D**). At 1200 μM, both δ- and γ-T3 revealed significantly higher GLUT2 expression than both negative control and glyburide (*p* < 0.05). δ-T3 enhanced a twofold higher GLUT2 expression compared to δ-T3 at 150 μM (*p* < 0.05) and 1.5 fold greater gene expression compared to δ- T3 at 300 μM (*p* < 0.05), respectively. δ-T3 also presented significantly higher GLUT2 expression than α-T3 at 150 μM (1.5 fold; *p* < 0.05), 300 μM (twofold; *p* < 0.05) and 1200 μM (1.8 fold; *p* < 0.05), respectively. As comparing δ-T3 to γ-T3, significantly higher GLUT2 expression was observed at 150 μM (1.5 fold; *p* < 0.05), 300 μM (1.9 fold; *p* < 0.05), 600 μM (1.8 fold; *p* < 0.05) and 1200 μM (1.4 fold; *p* < 0.05), respectively. The data demonstrated the synergistic activation of INS1 and GLUT2 gene expressions by δ- and γ-T3 treatments.

### Relative Gene Expression of Insulin Gene Transcription Factor

#### PDX1

**FIGURE 6:**
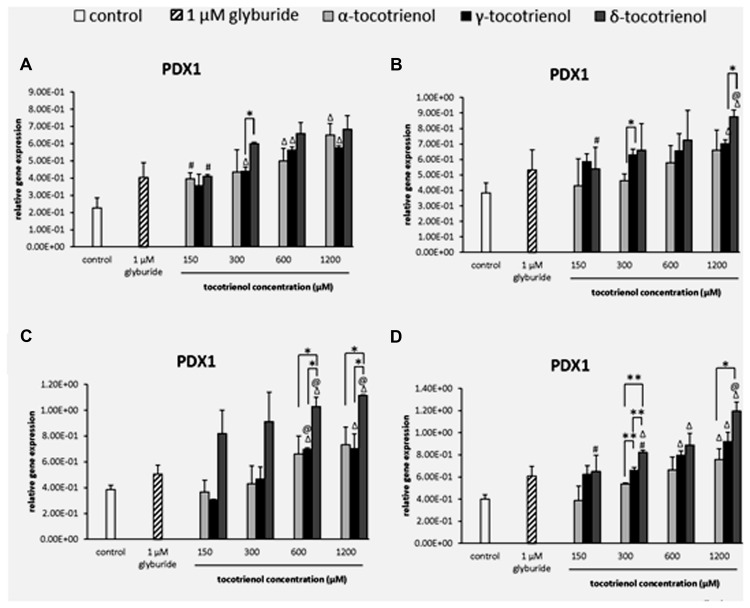
**The relative gene expression level of PDX1 in α-, γ-, and δ- tocotrienol treatment. (A)** 2.8 mM glucose: all δ-T3 groups are 0.05 relative to negative control, ^Δ^*p* < 0.05 relative to negative control, ^#^*p* < 0.05 relative to 1200 μM (same tocotrienol group), *p < 0.05 relative to matched group. **(B)** 2.8 mM glucose and 30 mM KCl (calcium raising agent): ^Δ^*p* < 0.05 relative to negative control, ^@^*p* < 0.05 relative to positive control, ^#^*p* < 0.05 relative to 1200 μM (same tocotrienol group), *p < 0.05 relative to matched group. **(C)** 16.7 mM glucose (stimulant): Δ*p* < 0.05 relative to negative control, ^@^*p* < 0.05 relative to positive control, *p < 0.05 relative to matched group. **(D)** 16.7 mM glucose and 30 mM KCl: ^Δ^*p* < 0.05 relative to negative control, ^@^*p* < 0.05 relative to positive control, ^#^*p* < 0.05 relative to 1200 μM (same tocotrienol group), *p < 0.05 and **p < 0.01 relative to matched group.

Based on the result of PDX1 gene expression, the effects of all three T3s were comparable with minimal effects on the PDX1 gene activation in basal glucose group. However, all three T3 were able to boost the gene expression at 600 and 1200 μM higher than the negative control (*p* < 0.05). Only α- and δ-T3 showed a significant increase of PDX1 gene expression in a dose dependent manner (*p* < 0.05). At 300 μM, δ-T3 exhibited 1.4 fold higher PDX1 expression compared to γ-T3 (**Figure 6A**). On the other hand, only δ-T3 showed greater treatment with up regulated gene expression than glyburide at 1200 μM with the addition of KCl (**Figure 6B**). Similar result was reported, indicating the less effectiveness of KCl role in basal glucose. While the effect of α-T3 was not as noble as δ- and γ-T3, both δ- and γ-T3 demonstrated strong stimulatory effect at 600 and 1200 μM with significantly higher PDX1 expression compared to glyburide in the stimulatory glucose condition (**Figure 6C**). At 600 and 1200 μM, δ-T3 showed significant greater gene expression compared to both negative and positive controls as well as to α- and γ-T3. At 600 μM, PDX1 expression at δ-T3 was increased to 1.5 fold compared to α-T3 (*p* < 0.05) and 1.3 fold compared to γ-T3 (*p* < 0.05). At 1200 μM, PDX1 expression in δ- T3 revealed 1.3 fold higher than α-T3 (*p* < 0.05) and 1.6 fold higher than γ-T3 (*p* < 0.05), respectively. The presence of KCl in the stimulatory glucose condition further enhanced the effect of δ-T3 in PDX1 expression in a dose dependent manner with a significantly higher expression compared to glyburide at 1200 μM (*p* < 0.05; **Figure 6D**). Significantly higher PDX1 gene expression was observed in all δ-T3 groups except at 150 μM when compared to the negative control (*p* < 0.05). A dose dependent increase in expression was observed in δ-T3 group (*p* < 0.05) where at 1200 μM it showed 1.8 and 1.5 fold increase compared to 150 and 300 μM, respectively. At 1200 μM, δ-T3 yielded a greater PDX1 activation compared to glyburide. For γ-T3, 600 and 1200 μM treatment produced significantly higher PDX1 expression compared to the negative control, while only α-T3 at 1200 μM revealed significantly higher PDX1 expression compared to the negative control. δ-T3 showed significantly effective treatment than α-T3 in up regulating the expression of PDX1 at 300 and 1200 μM. PDX1 expression was upregulated significantly in δ-T3 at 300 μM by 1.5 fold (*p <* 0.01) compared to α-T3 at 300 μM and 1.6 fold increase in δ-T3 at 1200 μM compared to α-T3 at 1200 μM (*p <* 0.05). At 300 μM, δ- T3 was found significantly better in upregulating PDX1 expression by 1.2 fold as compared to γ-T3 (*p <* 0.05). The strong stimulatory effect of δ-T3 in PDX1 gene activation was observed in basal and stimulant glucose condition. In contrast, the effect of α- and γ-T3 was not as noble as δ-T3. The effect of δ-T3 was greater than glyburide in high glucose as well as in high glucose with KCl suggesting KCl enhanced the effect of δ-T3 in PDX1 expression in a dose dependent manner.

#### MafA

**FIGURE 7:**
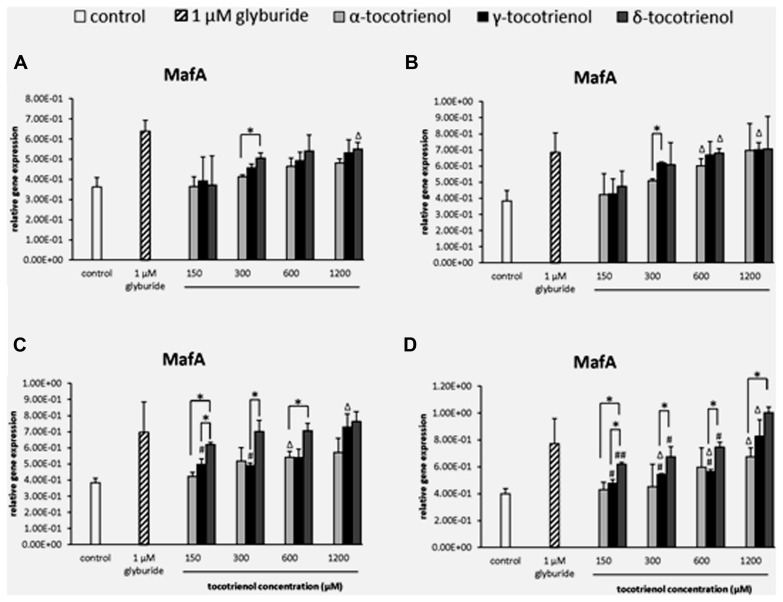
**The relative gene expression level of MafA in α-, γ-, and δ-tocotrienol treatment. (A)** 2.8 mM glucose: ^Δ^*p* < 0.05 relative to negative control, *p < 0.05 relative to matched group. **(B)** 2.8 mM glucose and 30 mM KCl (calcium raising agent): ^Δ^*p* < 0.05 relative to negative control, *p < 0.05 relative to matched group. **(C)** 16.7 mM glucose (stimulant): all δ-T3 groups are 0.05 relative to negative control, ^Δ^*p* < 0.05 relative to negative control, ^#^*p* < 0.05 relative to 1200 μM (same tocotrienol group), *p < 0.05 relative to matched group. **(D)** 16.7 mM glucose and 30 mM KCl: all δ-T3 groups are 0.05 relative to negative control, ^Δ^*p* < 0.05 relative to negative control, ^#^*p* < 0.05 and ^##^*p* < 0.01 relative to 1200 μM (same tocotrienol group), *p < 0.05 relative to matched group.

Similar to PDX1, all T3s displayed minimal effect on MafA gene expression in the basal 2.8 mM glucose group (**Figure 7A**) and basal with KCl group (**Figure 7B**) indicated the less effectiveness of KCl role in basal glucose. In contrast, δ- T3 had proven a significantly better treatment than γ- and α-T3 in the stimulatory glucose condition (**Figure 7C**). δ-T3 treatment revealed a significant improved of MafA expression with all four δ-T3 groups showed significant increase in expression compared to negative control (*p* < 0.05). However, only 600 μM of α -T3 and 1200 μM of γ-T3 presented a stimulatory effect on MafA expression with significant higher gene expression than the negative control (*p* < 0.05). δ-T3 was proven a better treatment than α-T3 at 150 and 600 μM: increased MafA expression by 1.5 fold at δ-T3, 150 μM (*p* < 0.05) and 1.3 fold at δ-T3, 600 μM (*p* < 0.05) compared to α-T3, respectively. While significantly higher MafA expression of δ-T3 were observed at 150 μM (1.2 fold; *p* < 0.05) and 300 μM (1.4 fold; *p* < 0.05) when compared to γ-T3. Moreover, both δ- and γ-T3 revealed better potency than α-T3 on MafA expression in the presence of KCl (**Figure 7D**). The addition of KCl in the incubation medium further enhanced the effect of δ- and γ-T3 in a dose dependent manner. δ-T3 treatment groups showed significant increase of MafA expression than the negative control (*p* < 0.05), The highest MafA gene expression in δ-T3 was observed at 1200 μM with significantly higher expression than at 150 μM (1.6 fold; *p* < 0.01), 300 μM (1.5 fold; *p* < 0.05) and 600 μM (1.3 fold; *p* < 0.05), respectively. Comparison between the effect of δ-T3 and α-T3 on MafA expression showed significant difference in gene expression at 150 μM (1.4 fold; *p* < 0.05) and 1200 μM (1.5 fold; *p* < 0.05), while the adverse effect of δ-T3 was also observed when compared to γ-T3. All four δ-T3 groups revealed significantly higher MafA expression compared to γ-T3 at 150 μM (1.3 fold; *p* < 0.05), 300 μM (1.2 fold; *p* < 0.05), 600 μM (1.3 fold; *p* < 0.05), and 1200 μM (1.2 fold; *p* < 0.05), respectively. δ-T3 possessed a better potency than α- and γ-T3 on MafA expression in the presence of high glucose concentration.

#### BETA2

**FIGURE 8:**
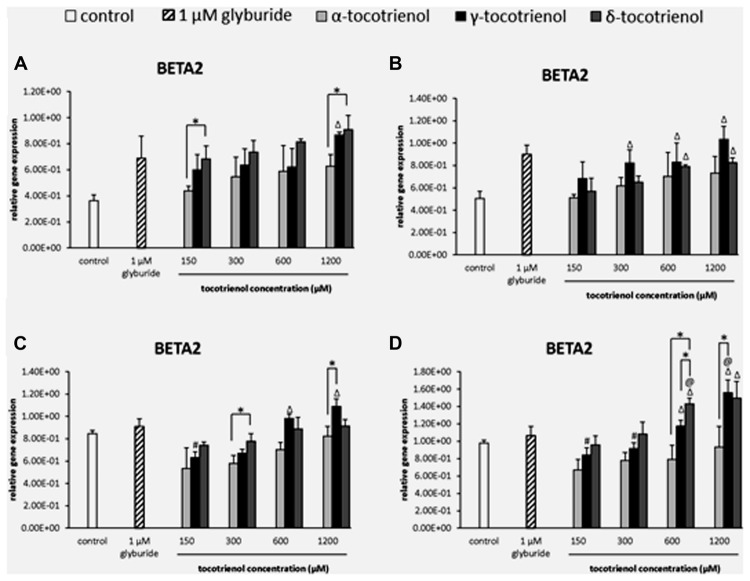
**The relative gene expression level of BETA2 in α-, γ-, and δ-tocotrienol treatment. (A)** 2.8 mM glucose: all δ-T3 groups are 0.05 relative to negative control, ^Δ^*p* < 0.05 relative to negative control, *p < 0.05 relative to matched group. **(B)** 2.8 mM glucose and 30 mM KCl (calcium raising agent): ^Δ^*p* < 0.05 relative to negative control. **(C)** 16.7 mM glucose (stimulant): ^Δ^*p* < 0.05 relative to negative control, ^#^*p* < 0.05 relative to 1200 ^μ^M (same tocotrienol group), *p < 0.05 relative to matched group. **(D)** 16.7 mM glucose and 30 mM KCl: ^Δ^*p* < 0.05 relative to negative control, ^@^*p* < 0.05 relative to positive control, ^#^*p* < 0.05 relative to 1200 μM (same tocotrienol group), *p < 0.05 relative to matched group.

The analysis of BETA2 gene expression showed that δ-T3 provided a better effect on gene expression compared to α-T3 in basal glucose incubation medium (**Figure 8A**). At 150 and 1200 μM, δ-T3 revealed a significant higher gene expression compared to α-T3 (*p* < 0.05): by 1.4 fold at 150 μM and by 1.6 fold at 1200 μM. However, the addition of KCl did not give a great impact on BETA2 gene expression as no activation of BETA2 expression was observed in α-T3 while only 600 and 1200 μM of both γ- and δ-T3 as well as γ-T3 at 300 μM showed significantly higher gene expression compared to the negative control (*p* < 0.05; **Figure 8B**). In contrast, the effect of δ-T3 was boosted in stimulatory glucose condition showing a significantly higher gene expression than α-T3 in a dose dependent manner (**Figure 8C**). While both δ- and γ-T3 activated BETA2 expression in stimulatory glucose condition with KCl. γ-T3 also showed significantly higher gene expression than the negative control at 600–1200 μM (*p* < 0.05) while only δ-T3 at 300 μM revealed a significantly higher BETA2 expression as compared to α-T3 at 300 μM (*p* < 0.05). In contrast, both δ- and γ-T3 activated BETA2 expression in stimulatory glucose condition with KCl (**Figure 8D**). At 1200 μM, γ-T3 significantly upregulated the expression of BETA2 as compared to both negative and positive controls (*p* < 0.05) as well as α-T3 (1.7 fold; *p* < 0.05). At 600 μM, δ-T3 showed a significant higher gene expression when compared to the positive control (*p* < 0.05), α-T3 (1.8 fold; *p* < 0.05) and γ-T3 (1.2 fold; *p* < 0.05). The study revealed that KCl had effectively enhanced the effect of δ-T3 at lower dosage (600 μM) and γ- T3 at higher dosage (1200 μM) with significantly higher expression of BETA2 than glyburide.

## Discussion

From the analysis of our findings, this study showed pharmacological activation of PPARδ and PPARγ by T3s (δ- and γ-T3) especially in the presence of high glucose concentration. PPARγ has been identified as the receptor for thiazolidinedione, an oral antidiabetic drug (Rosen et al., 2003) while there is no report of the PPARδ involvement with current antidiabetic drug yet. Nonetheless, it functions as transcriptional regulator for glucose homeostasis and lipid metabolism which is proven to act as a lipid sensor and is a potential therapeutic target to treat metabolic disease (Reilly and Lee, 2008) and regulated glucose metabolism and insulin sensitivity (Lee et al., 2006). However, the outcome from molecular docking revealed the minimal chance of direct interaction between these T3 derivatives and the ligand-modulated transcription factors. Together with the RTPCR analysis, it appears that the activation of PPARδ and PPARγ in parallel with other insulin genes transcription is able to regulate preproinsulin mRNA levels. Based on our findings, glyburide in contrast did not give great impact on glucose-stimulated proinsulin biosynthesis or preproinsulin mRNA levels although it is able to stimulated insulin secretion at both basal and stimulatory glucose concentrations, which is consistent with the result from previous studies (Levy and Malaisse, 1975; Wicksteed et al., 2003).

The smallest molecule of δ-T3 with greatest mobility and agility enabled a better penetration and interaction with β cell, as supported by Vignini et al. (2011). α-T3 on the other hand, is the least active form among all the T3 derivatives. The presence of KCl in the culture further increased the depolarization of the β cell membrane (Ren et al., 2007), facilitating the intake of glucose and T3 derivatives into the cells. This explains the better bioactivity of δ -T3 not only in the activation of PPARs, but also in the upregulation of other insulin secretion-associated gene expression levels. Together with previous findings, the high levels of T3s found in golgi apparatus and endoplasmic reticulum suggested that the ability of T3s to enhance the synthesis of preproinsulin in the endoplasmic reticulum and assisted the transport of proinsulin through golgi apparatus in the insulin synthesis and secretion process (Drevon, 1991; Ren et al., 2007). This finding supports the outcome of the up regulated gene expression level of PPARδ and PPARγ by δ- and γ-T3 in the stimulatory glucose groups but not α-T3 due to the unresponsiveness in the activation of PPARγ in stimulatory glucose groups. The upregulation in the PPARδ and PPARγ gene expression suggested that δ- and γ-T3 targeted at the same receptor which involve in the same mechanism in controlling glucose homeostasis. Nevertheless, further investigation such as luciferase assay needs to be carried out to identify the pathways involved.

**FIGURE 9:**
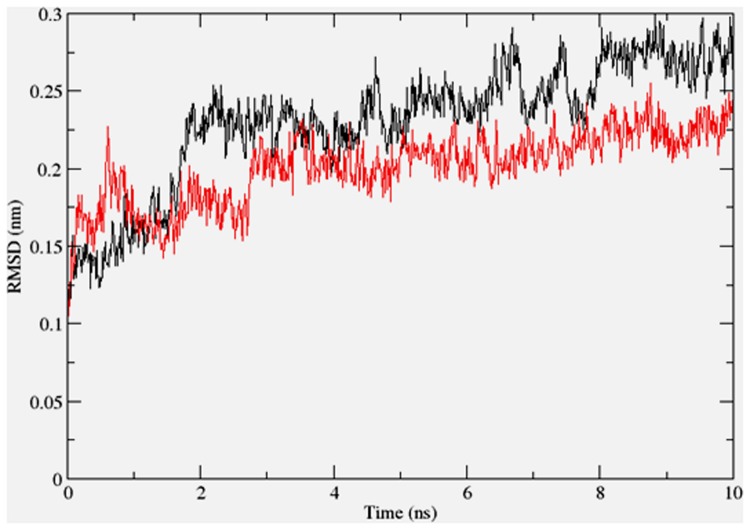
**RMSD plots of PPARδ backbone in complex with α-tocotrienol (black line) and δ-tocotrienol (red line) as a function of simulation time**.

**FIGURE 10:**
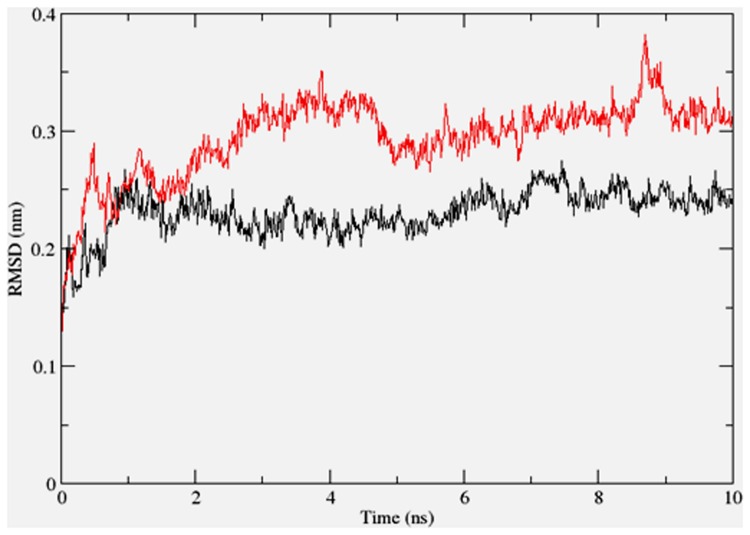
**RMSD plots of PPARγ backbone in complex with α-tocotrienol (black line) and δ-tocotrienol (red line) as a function of simulation time**.

**FIGURE 11:**
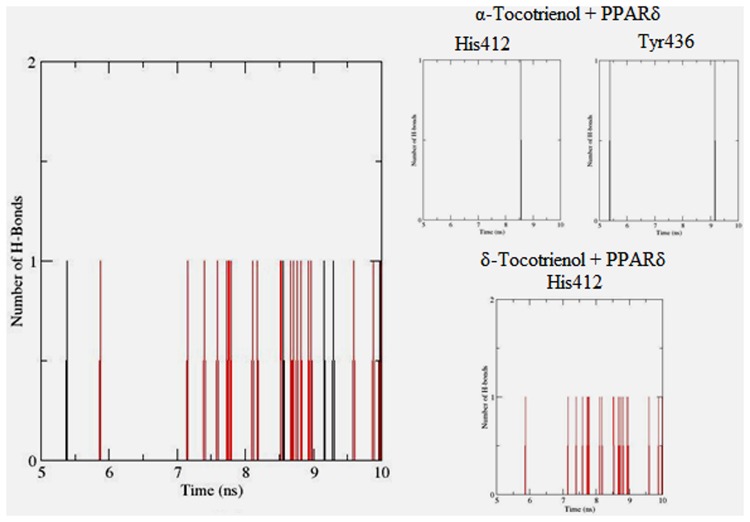
**Hydrogen bond plots between PPARδ residues and α-tocotrienol (black line) and δ-tocotrienol (red line) during the final 5 ns of simulation**.

**FIGURE 12:**
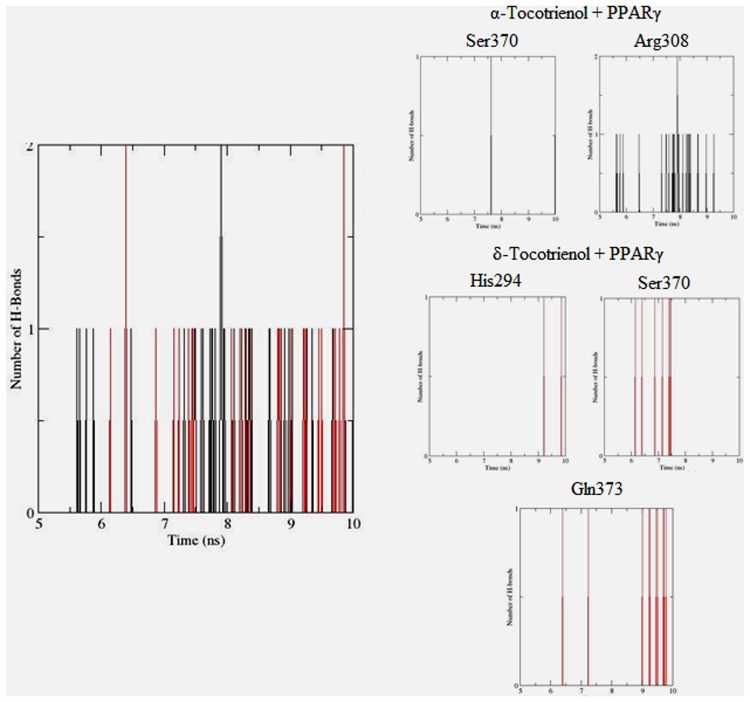
**Hydrogen bond plots between PPARγ residues and α-tocotrienol (black line) and δ-tocotrienol (red line) during the final 5 ns of simulation**.

**FIGURE 13:**
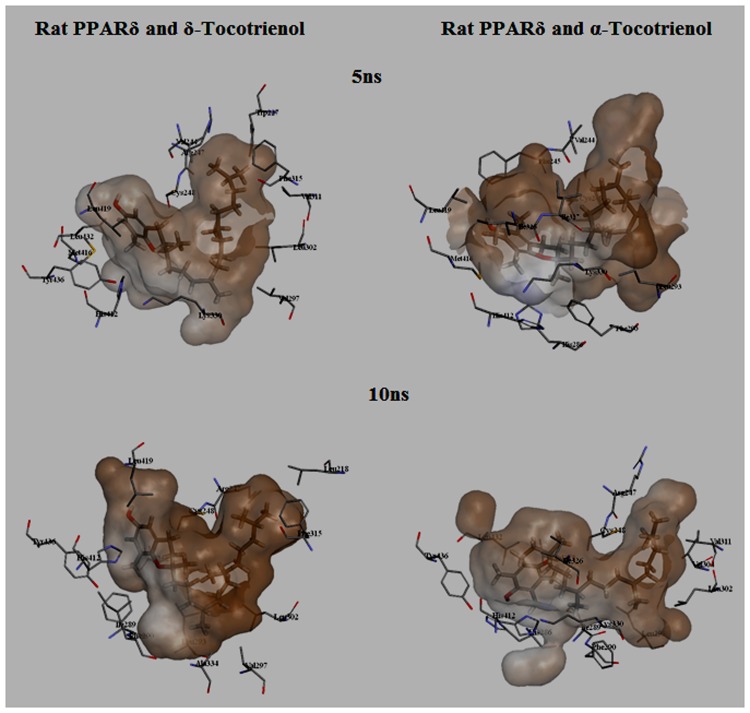
**MD simulation snapshots of δ-tocotrienol and α-tocotrienol complexed with rat PPARδ.** The atom coloring for δ-tocotrienol and α-tocotrienol are as follows: carbon in gray, oxygen in red, nitrogen in blue, sulfur in yellow, and hydrogen in white. The surface is colored by the hydrophobicity of the protein residues, from blue for hydrophilic to brown for hydrophobic.

**FIGURE 14:**
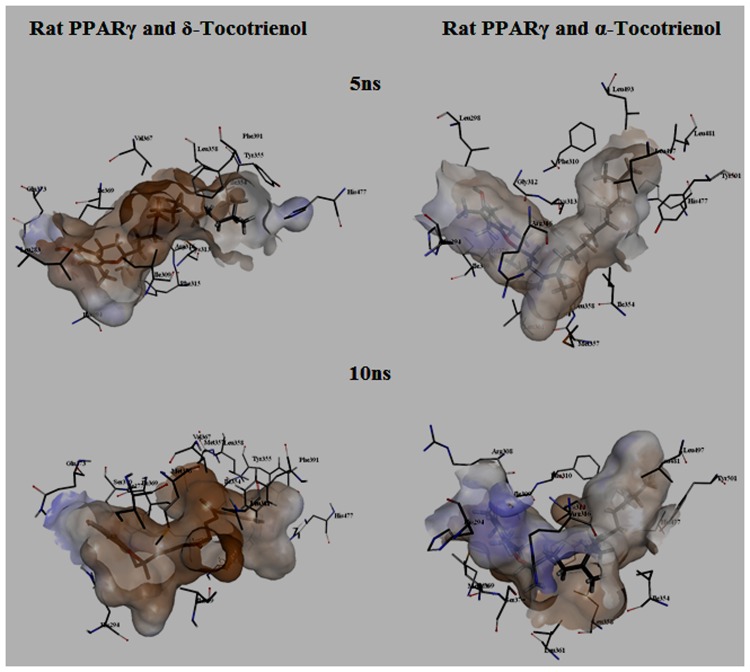
**MD simulation snapshots of δ-tocotrienol and α-tocotrienol complexed with rat PPARγ.** See **Figure 13** for legend.

To gain insights on how α- and δ-T3 could bind to rat PPARδ and PPARγ receptors, homology models were built from the crystal structures of human PPARδ (PDB id: 3GZ9) and PPARγ (PDB id: 1ZGY). These two T3s were specifically chosen for the docking study based on their effects on relative gene expression of ligand-modulated transcription factor level and other reported work (Fang et al., 2010). The compounds were docked to the plausible binding sites of the models as suggested by some of the literature reviews (Fratev et al., 2015; Maltarollo et al., 2015). First, the cDOCKER program generated more than 100 binding poses. To choose the best conformation, the protein–ligand binding interactions were visually inspected followed by ranking them according to their binding interaction energies and clustered according to their binding conformations. The best ligand–protein complexes were subjected to molecular dynamics simulation using GROMACS 5.0.4. As shown in **Figures 9** and **10**, all protein–ligand complexes reached equilibrium state after 10 ns of simulation as shown in the RMSD plots of the protein backbone of PPARδ and PPARγ in complex with α- and δ-T3. There was no major fluctuation in both the RMSD of PPARs backbone during the course of 10 ns simulation suggesting that the binding of α- and δ-T3 did not induce any major changes to the protein conformation. In term of H-bonding interaction, δ-T3 showed higher affinity to form H-bonding interaction with His412 compared to α-T3 which recorded only a single interaction with PPARδ during the course of simulation (**Figure 11**). This could be the key reason why δ-T3 could activate PPARδ but not α-T3 as reported by Wong and co-workers (Fang et al., 2010). Future development of δ-T3 into a potent PPARδ agonist should focus on this region of interest. On the other hand, δ-T3 could form three H-bonds with His294, Ser370 and Gln373 residues of PPARγ while α-T3 predominantly engaged with Arg308 (**Figure 12**). **Figures 13** and **14** summarize the overall non-polar interactions of δ-T3 and α-T3 with the residues in rat PPARδ and PPAR**γ** during the final 5 ns of simulations.

The significantly higher INS1 gene expression in the stimulatory glucose groups showed that δ-T3 was able to enhance preproinulin mRNA synthesis level while γ- and α-T3 showed less impact on the transcriptional level. Metabolic regulation of insulin gene expression by δ-T3 enabled the maintenance of sufficient intracellular insulin in β-cell to sustain the secretory demand in high glucose condition (Poitout et al., 2006). Short-term regulation of insulin biosynthesis transcription and translation level as reported by Leibiger et al. (1998) supported our outcome where a short term exposure of isolated pancreatic islets to stimulatory glucose (16.7 mM) resulted in a 2- to 5-fold elevation in (prepro)insulin mRNA levels within 60–90 min. This finding is further supported by the short half-life and rapid turnover of T3 derivatives, by Yap et al. (2003). The cell culture microenvironment provided by the microfluidic device further enhanced the lifespan of the β cells and consistencies of the treatment throughout the 1 h treatment (Hung et al., 2005). PPARγ has also been reported to significantly activate GLUT2 in the rat β cells (Higa et al., 1999) supporting GLUT2 expression in parallel with this enhanced response. δ- T3 activated GLUT2 in stimulatory glucose condition and further improved by the administration of KCl, which in turn enhanced glucose entrance into β cells (Vaulont et al., 2000). The synergistic effects of both INS1 and GLUT2 gene in δ- and γ-T3 treatment revealed that glucose played a major role in the gene expression despite of the effectiveness of the T3.

PDX1 is the most important insulin transcription factor in glucose-stimulated insulin gene transcription and is only expressed in pancreatic β cell (Ren et al., 2007). From our findings, δ-T3 was able to upregulate the expression level of PDX1 to about twofold in both basal and stimulatory glucose groups with added KCl at 1200 μM compared to the negative control, where similar research outcome has been reported by Mosley and Ozcan (2004). A significantly upregulated expression of MafA in both stimulatory glucose as well as stimulatory glucose with KCl group in δ- and γ-T3 showed that exposure of β cells to both glucose stimulant group had significantly increased MafA mRNA expression which is also supported by Hagman et al. (2005). Moreover, both δ- and γ-T3 displayed dose dependent manner that possessed better potencies than α-T3 on MafA expression in the presence of KCl. The similarity on the effects of each T3 derivatives on these insulin gene transcription factors (PDX1, MafA, and BETA2) showed the synergistic effect of these genes in insulin transcription and mechanism (Zhang et al., 2005).

While α-T3 and glyburide exert no activating effect, a significantly upregulated BETA2 gene expression was observed at δ-T3 (600 μM) and γ-T3 (1200 μM) in stimulatory glucose group with added KCl compared to glyburide strongly supported the effect of δ- and γ-T3 in the regulation of insulin gene expression. MafA activates PDX1 and BETA2 in established mechanism in glucose-stimulated insulin secretion pathway (Ren et al., 2007). Interestingly, the upregulated gene expression of MafA, PDX1, and BETA2 by δ- and γ-T3 at stimulatory glucose condition and added KCl confirmed the effects of both T3 derivatives in the enhancement of targeted activation of insulin gene transcription. The effects of δ-T3 in stimulatory glucose with KCl are confirmed with statistically significant of PPARγ, GLUT2, PDX1, and BETA2 gene expression levels compared to glyburide. δ-T3 was able to enhance insulin synthesis in response to glucose and KCl whereas α- and γ-T3 having less potency than δ-T3 at similar concentrations. Therefore, the overall results suggest that δ-T3 exhibited a greatest potency in the activation of the insulin transcriptional factors. Since the targeted pancreatic β cell involved in the insulin synthesis are responsible for relative deficiency of insulin secretion that leads to type 2 diabetes mellitus, the findings of the study have provided the important role of δ-, γ-, and α-T3 in the prevention of hyperglycemia as well as the medical management of glucose homeostasis.

## Conclusion

In summary, we have discovered the essential role of δ-, γ-, and α-T3 in the mechanisms to enhance insulin release through activation of insulin secretion-associated genes expression. The study was conducted in the microfluidic device which was able to provide a continuous supply of nutrient and maintenance of the accuracy of tocotrienol dosage throughout the treatment. The outcome could have given a remarkable impact on the cell culture research. The gene expression analysis in tocotrienol treatments suggested an important role of tocotrienol derivatives in the activation of insulin transcription factors following an impact on insulin secretion. Apart from the effectiveness of tocotrienols, glucose played a major role in the gene expression study where most of the insulin secretion associated genes were expressed depending on the presence of high glucose (16.7 mM). In addition, δ-T3 revealed the greatest impact on insulin synthesis level in the glucose-stimulated insulin secretion pathway than γ- and α-T3. δ-T3 together with the presence of membrane depolarising agent, KCl in the stimulatory glucose cell culture induced greatest insulin synthesis suggesting a synergistic effect of δ-T3 and KCl in the regulation of insulin gene transcriptional level in the hyperglycemic condition. Our discovery has also provided new prospects for the medical management of hyperglycemia and glucose homeostasis associated with type 2 diabetes mellitus.

## Author Contributions

IJ, LC, and KC participated in the design and coordination of the study, analysis, and interpretation of the data. IJ edited the manuscript and gave the final approval of the version to be submitted for publication. LC carried out the experiments, analyzed the data and drafted the manuscript. KL, KR, and MA were involved in the molecular docking experiments.

## Conflict of Interest Statement

The authors declare that the research was conducted in the absence of any commercial or financial relationships that could be construed as a potential conflict of interest. The reviewer MB and handling Editor declared their shared affiliation, and the handling Editor states that the process nevertheless met the standards of a fair and objective review.

## Footnotes

1 www.ncbi.nlm.nih.gov
